# The Exterior of the Horse

**Published:** 1892-03

**Authors:** 


### REVIEWS.

The Exterior of the Horse. By Armand Goubaux and Gustave Barrier. Translated and Edited by Simon J. J. Harger, V. M. D.

In most branches of science the movement toward a systematic statement and collection of facts is slow. Detached information, traditions, and quackeries lie heaped together in odd volumes, old pamphlets, inaccessible periodicals, and, worse still, in the unstable minds of investigators. Some day an energetic thinker comes along, gathers up all the learned *debris*, puts it into order, collates it, and expresses from it its essential conclusions. Then we have a standard work on a given subject. This is the process through which the material in "The Exterior of the Horse," by MM. Armand Gobaux and Gustave Barrier, has gone before passing into the handsome and substantial volume of 916 pages now before us.

In France, where this great book was issued first in 1884, it is the recognized authority on the external aspect of the horse, in so far as that affects his mechanical aptitudes and his commercial value. This side of the subject had previously been much obscured by theory and empirical treatment, but the authors started out with a directness of aim and a thoroughness of knowledge which withheld them from following the lead into obscurity. They made a work which has a practical value for the horseman and the breeder, as well as a scientific interest for the practitioner and the student. Indeed, to the veterinarian, the student, and the horseman, the book absolutely invaluable.

To give an example of its exhaustive character, it may be stated that, after a preliminary study of animal mechanics, the head alone is considered through sixty pages, the body through eighty-eight, and the members through one hundred and fifty. Each of these departments has a wealth of illustrations and of practical detail which makes clear even to the most unenlightened amateur all that need be known. Section third is devoted to Proportions, and goes back to the earliest historical sources, namely, Abou Bekr, and the Italian Grisone, for its interesting data, through which the study of equine beauties and graces becomes fascinating as well to the layman as to the veterinarian.

The fourth section treats of the horse in relation to locomotion, and includes in its wide-reaching investigations an exposition of Prof. Muybridge's photographic experiments with the horse in motion. Then there are other sections, up to the eighth, in which is given the same careful information on the age and how to determine it by the teeth ; on the coats, and the height ; on the services of race-horses, horses of luxury, cavalry horses, and horses of industry and commerce ; on vicious horses and their habits ; and, finally, there is good advice on the choice of a horse to both seller and purchasers. The mere statement that the volume contains three hundred and forty-six illustrations is quite sufficient to show what cost and care have been involved in its production.

For all these benefits, the very large part of the community interested in the horse owes a debt of gratitude to the translator, Prof. Simon J. J. Harger, V. M. D., of the University of Pennsylvania, who has done his work admirably and thororughly. Dr. Harger, as a student, showed a marked predilection and ability for the study of anatomy, and from the day of his graduation, devoted himself to teaching, first, as demonstrator of anatomy, and later as Professor of Anatomy and Zootechnics. We will hope to see him add more to our veterinary literature. The publishers, the J. B. Lippincott Co., have spared no pains or expense in bringing forth a work which will undoubtedly become a standard here, as it is already in France, in the college as well as in the stable.

